# HIV and Drug-Resistant Subtypes

**DOI:** 10.3390/microorganisms11010221

**Published:** 2023-01-15

**Authors:** Bianca Maria Nastri, Pasquale Pagliano, Carla Zannella, Veronica Folliero, Alfonso Masullo, Luca Rinaldi, Massimiliano Galdiero, Gianluigi Franci

**Affiliations:** 1 Department of Experimental Medicine, University of Campania “Luigi Vanvitelli”, 80138 Naples, Italy; 2 Department of Medicine, Surgery and Dentistry “Scuola Medica Salernitana”, University of Salerno, 84081 Baronissi, Italy; 3 Infectious Disease Unit, San Giovanni di Dio e Ruggi D’Aragona University Hospital, 84125 Salerno, Italy; 4 Department of Advanced Medical and Surgical Sciences, University of Campania Luigi Vanvitelli, 80138 Naples, Italy; 5 Clinical Pathology and Microbiology Unit, San Giovanni di Dio e Ruggi D’Aragona University Hospital, 84125 Salerno, Italy

**Keywords:** HIV, ART, drug resistance, retrovirus, NNRTIs, NRTIs, PIs, INSTIs, entry inhibitors, AIDS

## Abstract

Acquired Immunodeficiency Syndrome (AIDS) is a human viral infectious disease caused by the positive-sense single-stranded (ss) RNA Human Immunodeficiency Virus (HIV) (Retroviridae family, Ortervirales order). HIV-1 can be distinguished into various worldwide spread groups and subtypes. HIV-2 also causes human immunodeficiency, which develops slowly and tends to be less aggressive. HIV-2 only partially homologates to HIV-1 despite the similar derivation. Antiretroviral therapy (ART) is the treatment approved to control HIV infection, based on multiple antiretroviral drugs that belong to different classes: (i) NNRTIs, (ii) NRTIs, (iii) PIs, (iv) INSTIs, and (v) entry inhibitors. These drugs, acting on different stages of the HIV life cycle, decrease the patient’s total burden of HIV, maintain the function of the immune system, and prevent opportunistic infections. The appearance of several strains resistant to these drugs, however, represents a problem today that needs to be addressed as best as we can. New outbreaks of strains show a widespread geographic distribution and a highly variable mortality rate, even affecting treated patients significantly. Therefore, novel treatment approaches should be explored. The present review discusses updated information on HIV-1– and HIV-2–resistant strains, including details on different mutations responsible for drug resistance.

## 1. Introduction: HIV and Its Distribution

AIDS is a global pandemic that currently affects approximately 38 million people worldwide. It is caused by two different lentiviruses, HIV-1 and HIV-2, that derived from multiple cross-species transmissions of the simian immunodeficiency virus until they affected humans [1].

The first isolation of the main agent of AIDS, HIV, was reported in 1983 by Robert Gallo [2] and Luc Montagnier [3], but several studies have documented that the HIV-1 virus was already present in Africa during the 1950s and America during the 1970s, and it spread all over the world in the following years with tens of millions of cases [4].

Two years later, in 1985, HIV-2 was identified as partially responsible for the AIDS pandemic but causing a milder form of immunodeficiency; data about HIV-2 are still limited, and only a few subtypes have been described.

**Figure 1:**
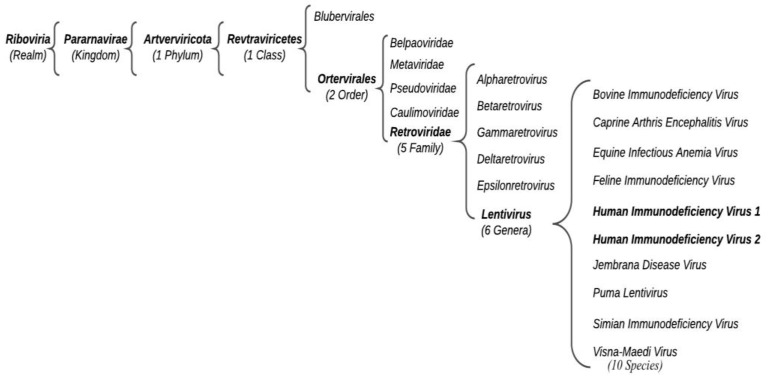
Schematic representation of HIV current classification.

According to the International Committee for the Taxonomy of Viruses (ICTV), HIV belongs to the *Retroviridae* family, *Orthoretrovirinae* subfamily, and *Lentivirus* genus (Figure 1).

Based on different genome sequences risen from an independent cross-species transmission event, we can distinguish HIV-1 groups M (major), O (outlier), N (non-M, non-O), and P.

Group M is the most widespread and includes nine subtypes: A, B, C, D, F, G, H, J, and K. The genetic distance between the subtypes is lower than the one across the groups [5,6,7,8,9].

Moreover, 19 circulating recombinant forms (CRFs) have been identified, including some that play important roles in the epidemic trend [8].

A recombinant is a genetic sequence that carries regions from two genetically distinct parental strains. It depends on the combination of viral genomes of different subtypes affecting the same cell. This leads to multiple changes in its genome, which results in regions from each different subtype [9,10]. The recombination phenomenon increases overall, i.e., the genetic complexity of viral populations more than just the accumulation of site mutations, allowing viral diversification to evade the host immune system and antiretroviral treatment [9].

In addition, the different subtypes have distinct global distribution patterns [5,11].

Therefore, it is clear how the genetic variability of HIV-1 can play a crucial role in its pathogenesis, transmission, diagnosis, treatment, and vaccine development, as well as in HIV drug resistance (HIVdr); for example, group O viruses are known to be naturally resistant to non-nucleoside reverse-transcriptase inhibitors, and M subtypes have all similar susceptibilities to some antiretroviral drugs in use [12,13].

There is a well-defined geographical distribution throughout the world of genetically distinct viruses. In particular, subtype B has become prevalent in almost all parts of Europe, Australia, and North America; subtype C has been mostly found in India and Brazil, as well as South and East Africa; subtype E is common in Thailand; and subtype F has been reported in Romania, Brazil, and Russia [5,14,15]. A large variety of subtypes, such as those of group M, has been reported in Africa, especially in the western central part. Instead, HIV viruses belonging to groups N, O, and P have not spread widely [5]. In addition, HIV-2 cases have been reported especially in Western Africa, whereas only a few have been reported in Europe (Portugal and France), India, and the United States of America [16]. The HIV-2 groups have derived from independent transmissions from sooty mangabeys (*Cercocebus atys*) to humans. HIV-2 strains are classified into eight groups, named A to H, but only groups A and B can cause epidemics. Isolates from group A are responsible for most of the HIV-2 infections worldwide (especially in Guinea-Bissau), while isolates from group B have been found in Ivory Coast and Ghana. More than one recombinant form, containing sequences of groups A and B, has been identified in Japan, Cameroon, and Ivory Coast. These observations differ largely from those of the other groups since infection with groups C to G has been found in few individuals and rarely leads to immune suppression. Coinfections of HIV-1 and HIV-2 are not as common in West Africa, where they represent 0.3–1% of all HIV-infected patients [17].

## 2. HIV Diagnosis and Clinical Course

Since HIV was determined to be the pathogen causing AIDS, important improvements have been made to its detection and characterization [18]. However, much depends on the time after the exposure, which leads to infection, since there is a high probability that, during this time, there are no diagnostic tests capable of detecting HIV [19]. In general, HIV RNA is the first marker of infection, which appears to be detectable within 12 days, then reaches its peak around the 20th or 30th day post-infection; meanwhile, on the 15th day, the capsid protein p24 also reaches detectable levels [19].

The first HIV diagnostic test was approved by the US Food and Drug Administration (FDA) in 1985. Today, there are several diagnostic tests, but only the serological and molecular methods remain the gold standard for diagnosing and monitoring HIV infection. In particular, the serological test is used to detect HIV-specific antigens and antibodies, while the molecular assay monitors antiretroviral success and drug resistance [18]. 

The serological methods consist of using enzyme immunoassays to detect HIV antigens and antibodies, whereas molecular methods consist of amplifying viral RNA in vitro by PCR. Additionally, testing algorithms associated with the techniques used need to be improved for newer detection assays [20] since they can potentially decrease the total number of tests performed and reduce the time between the test and the initiation of treatment [21].

Recent studies highlight the importance of starting an early ART once HIV has been diagnosed [22]. Despite this, because of reasons still unknown but possibly associated with immune and genetic factors, some rare individuals do not need antiretroviral therapy thanks to a spontaneous control of viremia (SCV) [23].

In most cases, instead, HIV infections are treated with ART that, in most of the patients, leads to an acceptable virological suppression if started before any compromise of immune functions [24,25]. 

Of course, being able to detect HIV early remains the most important element to reduce disease advancement since diagnosis is followed by antiretroviral therapy that increases patient life expectancy and reduces the possibilities of transmission. 

## 3. Treatment

Before the discovery of antiretroviral therapy (ART), HIV/AIDS patients were initially treated with only one or two antiretroviral drugs, which belonged to the class of nucleotide analogs, whose abilities to reduce viral load were very low and hampered by the rapid insurgence of resistance and, consequently, were not able to modify the course of the disease. Approximately 23 years ago, ART, which is based on the combination of different classes of antiretroviral drugs, was developed. This treatment has significantly prolonged survival in patients with AIDS (about 7 to 10 years of survival or longer). The first drug to treat HIV/AIDS approved by the US Food and Drug Administration was zidovudine, a nucleoside reverse-transcriptase inhibitor (NRTI), in 1987, but studies highlighting the effectiveness of combined administration of two drugs NRTI were already released at the beginning of 1990 [26,27,28]. 

ART can include a combination of nucleoside reverse-transcriptase inhibitors (NRTIs), non-nucleoside reverse-transcriptase inhibitors (NNRTIs), protease inhibitors (PIs), integrase strand transfer inhibitors (INSTIs), and entry inhibitors.

Since these drugs belong to different classes, they have different mechanisms of action by which they interfere with a unique step in the life cycle of the virus. Generally, two NRTIs are used in association with another class, such as PI or INSTI, but current data highlight that a simplified two-drug treatment can be equally effective [29]. All these treatments can be modified based on toxicity and induced resistance.

NNRTIs and NRTIs inhibit the polymerase activities of HIV-1 reverse transcriptase (RT), which plays a pivotal role in the HIV life cycle. Reverse transcriptase (RT) is essential for the life cycle of HIV because it converts ingle-stranded genomic RNA into double-stranded DNA, which is subsequently integrated into the host chromosome and passed on to all progeny cells [30].

For that reason, HIV RT has been an ideal target for antiretroviral agents. NRTI drugs approved and used in ART are abacavir, didanosine, emtricitabine, lamivudine, stavudine, tenofovir, and zidovudine, while NNRTI drugs are doravirin, delavirdine, efavirenz, etravirine, nevirapine, and rilpivirine. Many of these drugs are not suggested by current guidelines, owing to several considerations regarding their toxicity or antiviral effects. 

Protease inhibitors (PIs) are a major class of drugs effective in treating HIV infection/AIDS because of their high effectiveness in inhibiting HIV replication and their relatively low resistance rate. In the past several years, a variety of protease inhibitors have been found [31]. As expected, these drugs interact with HIV-1 protease, which is responsible for the production of all viral enzymes and structural proteins necessary to produce mature, virulent virions, starting from long polypeptide chains, which are cut to obtain single proteins [32,33].

Therefore, the inhibition of the protease activity effectively interferes with a vital stage in the HIV life cycle [34]. PI drugs include atazanavir, darunavir, fosamprenavir, indinavir, nelfinavir, ritonavir, saquinavir, and tipranavir; presently, darunavir is the most frequently used.

The integrase (IN) is an enzyme that HIV uses to insert its viral DNA (vDNA) into the DNA of the host cell through two catalytic actions: 3′ processing and strand transfer.

Although the integrase strand transfer inhibitor (INSTI) antiretroviral drugs are the latest class of agents approved [34,35], they have a key role in the treatment of an HIV-positive individual by inhibiting the strand transfer step made by INs through competitive binding to the enzyme’s active site. Then, INSTIs stop IN action also by chelating the divalent cation (Mg^2+^ or Mn^2+^) that is required for its enzymatic activity [36].

There are currently four INSTIs approved for the treatment of HIV infection: raltegravir, elvitegravir, bictegravir, and dolutegravir [37].

Finally, entry inhibitors prevent the virus from entering the cell and include CCR5 antagonist and entry inhibitor of gp41. HIV-1 uses various membrane proteins as a receptor to bind the plasma membrane, such as CCR5 [38,39], or uses gp41, a glycoprotein that physically brings the viral envelope closer to that of the cell membrane, allowing their fusion. Maraviroc binds CCR5, while enfuvirtide binds gp41 [40], preventing the virus’s cell binding or induced membrane fusion and, consequently, its entry into the cell.

Other noteworthy and recent drug classes are capsid inhibitors and NNRTIs.

Lenacapavir represents a new class of drugs called capsid inhibitors [41], approved in December 2022 for the first time by the FDA. Its mechanism of action is inhibiting the disassembly of the capsid shell, a step essential for viral replication. It is used in association with other drugs.

NNRTIs, such as islatravir, which is still in phase 3 clinical trial, inhibit reverse transcription via multiple mechanisms, which can confer a unique mutant susceptibility profile [42].

The treatment for HIV-2 is not the same as the one for HIV-1; in fact, drugs used to treat HIV-2 are NNRTIs and entry inhibitors, while some PIs have weak or no inhibitory activity against HIV-2 [17].

Some people receiving ART have shown severe side effects, such as diarrhea, headache, fatigue, rash, or, worse, treatment failure. Nevertheless, ART has been revealed to be really successful and has allowed the majority of infected people to conduct their normal lives. 

The emergence of HIV drug-resistant subtypes, especially caused by the rapid and error-prone replication of the virus, is increasing [30].

Moreover, along with a growing number of drug-resistant strains, ART has led to an ever-growing pool of individuals who can transmit drug-resistant strains of HIV-1 [43,44].

## 4. Drug Resistance Mechanism

HIV-1 virus has a high mutation rate, accumulating nearly one nucleotide mutation per replication cycle (Table S1). That is the main reason why there is a considerable HIV-1 variation in patients [45,46].

Since people are usually infected with only a single or a few original clones [47], an exponential number of virions are likely produced each day in untreated individuals, resulting in innumerable variants of the virus. The high HIV-1 recombination rate, due to different variants infecting the same cell, together with its capacity of reactivation in infected cells, increases the complexity of this virus [48,49]. As a matter of fact, it is possible to find a wide spectrum of virus variants infecting the same patient. Unfortunately, most of the time, these variants gain new mechanisms to evade the immune system of the host [50]. 

People harboring HIV-resistant strains can be divided into two categories: people on ART that acquire HIV drug resistance (ADR) and people who become infected with HIV-resistant strains. 

Among people receiving ART, resistance can frequently develop, prompted by suboptimal drug treatment, which can lead to the selection of drug-resistant viruses [46]. When virus replication occurs in the presence of suboptimal concentrations of the drug, drug-resistant viruses are selected, and the replication of drug-resistant viruses in the presence of the drug can further increase the virus’ mutation rate [46].

Other than acquired drug resistance and transmitted resistance, naturally drug-resistant viruses are extremely rare when the viruses are not subjected to selective drug pressure, even more in untreated patients. HIV-1 genetic variability is ensued by the HIV-1 reverse transcriptase (RT) processing errors by recombination when more than one viral variant infects the same cell and by the accumulation of proviral variants during the course of infection [45,49,51,52]. Although most HIV-1 infections are initiated by a single viral variant [39], innumerable variants related to the initially transmitted virus emerge within weeks following infection [51,53].

There are often high levels of cross-resistance within each drug class. Most drug resistance mutations in a specific antiretroviral class decrease susceptibility to one or more antiretroviral drugs of the same class [54] (Table S1, Supplementary Materials). In contrast, viruses with high levels of drug resistance in one specific antiretroviral class are generally susceptible to drugs that belong to another class. However, there are few cases of cross-resistance between drug classes. Recently, Sun et al. demonstrated the prevalence of doravirine cross-resistance in HIV-infected adults who failed first-line ART [55]. Doravirine resistance was highly associated with efavirenz and nevirapine resistance and moderately with etravirine and rilpivirine resistance.

Several studies have recently shown an increase in primary resistance rates in various geographic areas [56,57], and a genotypic resistance test is now recommended before the initiation of ART in patients with HIV infection.

## 5. NRTI Drug Resistance

Nucleoside reverse-transcriptase inhibitors (NRTIs) form the backbone of ART [58].

NRTI drug resistance works in two different biochemical ways. The first mechanism relates to the RT enzyme’s ability to avoid the binding of the NRTI, while retaining the ability to recognize the natural dNTP substrates during polymerization. The second mechanism, instead, relates to the promotion of the hydrolytic removal of the chain terminating NRTI, continuing DNA synthesis [54].

Therefore, HIV has two major routes to escape nucleoside and nucleotide reverse-transcriptase inhibitor class drug selection pressure. One path is through the selection of thymidine analog mutations (TAMs; M41L, D67N, K70R, L210W, T215F/Y, and K219Q = E), which act to increase excision of NRTIs. Interestingly, the TAM pathway 1, which includes mutations at codons 41, 210, and 215, was prevalent among subtype B viruses, whereas mutations at codons 67, 70, and 219 were the most present among subtype C and F viruses. These mutations can be selected either by zidovudine (ZDV) or by stavudine and confer resistance to these drugs [30,59]. 

The second route is by the selection of mutations that allow the virus to fully distinguish the natural substrate from the modified one at the time of incorporation, such as M184I = V, L74V, and K65R in HIV-1 subtype B and C47. Several lines of evidence suggest that K65R is more likely to emerge in subtype C viruses than in viruses belonging to other subtypes. The K65R resistance pathway has a favorable resistance profile conferring low- to intermediate-level phenotypic resistance to most NRTIs. This mutation is often considered the tenofovir DF–resistance mutation, but it also works with other nucleoside analogs, including abacavir (ABC) and didanosine (ddI) [58,60,61,62,63]. However, clinical studies have shown a low incidence of K65R resistance in drug-naive and treatment-experienced patients [58,62,64,65].

Conversely, the presence of the M184V/I amino acid substitution, which is the hallmark of lamivudine (3TC) and emtricitabine (FTC) resistance, is associated with increased response to tenofovir DF [66]. There is no significant difference between B and non-B subtypes for tenofovir and emtricitabine/lamivudine drug resistance [67]. CRF01_AE viruses preferentially develop the NRTI-resistance mutation V75M58. Of note, it is still debated whether the association of abacavir–lamivudine–efavirenz is related to the development of drug resistance [68,69,70].

Reduced viral sensitivity to zidovudine largely results from an accumulation of mutations, with a mutation at codon 70 (K70R) emerging first, followed by codon 215 substitutions (T215Y or T215F) and mutations at codon 41(M41L) [71,72]. The combination of mutations 215 and 41 confers the highest level of zidovudine resistance. 

Finally, NRTI resistance in HIV-2 has a lower genetic barrier than HIV-1. In HIV-2, a common mutation is Q151M, which confers resistance to all NRTIs (especially to zidovudine) except tenofovir. There are also K65R, M184I, or V mutations frequently found in viral isolates that confer resistance to more than one NRTI. M184I and M184V confer high-level resistance to emtricitabine, whereas K65R confers resistance to tenofovir and emtricitabine [17].

## 6. NNRTIs Drugs Resistance

NNRTIs have a relatively low genetic barrier to resistance. In general, NNRTIs interact with a hydrophobic pocket within the HIV-1 reverse-transcriptase enzyme. Almost all NNRTI-resistance mutations are located in the RT domain hosting this binding pocket [73]. Since the NNRTI-resistance mutation reduces susceptibility to two or more NNRTIs and there are multiple independent NNRTI-resistant lineages, because of the low genetic barrier, there is a high level of cross-resistance inside this drug’s class [54,74,75,76].

As previously described, there is a significant difference between B and non-B subtype viruses. Moreover, non-B subtype viruses were found to have more resistance-associated mutations than B subtype viruses. This difference is mainly due to the widespread mutation E138A, which is twice as common among non-B subtype viruses than B subtype viruses [67].

However, the most common NNRTI mutations are L100I, K101E/P, K103N/S, V106A/M, Y181C/I/V, Y188C/H/L, G190A/S/E, and M230L. K103N is the most frequent NNRT-associated mutation. Each of these mutations, except for L100I, causes intermediate- or high-level phenotypic resistance to nevirapine and, with the exception of Y181C/I/V, efavirenz [77]. It has also been observed that they all cause phenotypic etravirine and rilpivirine resistance except for K103N/S, V106A/M, Y188C/H/L, and G190A/S/E64.

The NNRTI-resistance mutation V106M occurs more often in subtype C viruses from patients treated with nevirapine or efavirenz because V106M requires a single base-pair change in subtype C viruses—GTG (V) => ATG (M)—but a two base-pair change in all other subtypes—GTA (V) => ATG (M) [78].

HIV-1 subtype A viruses develop the NNRTI-resistance mutation G190S [79]. To develop high-level resistance, one mutation is required in the case of nevirapine, one to two in the case of efavirenz, and two in the case of etravirine [77,80,81]. Rilpivirine and etravirine have similar structures, but despite this, rilpivirine has a lower genetic barrier to resistance than etravirine because it is administered at a much lower dose [82,83]. Etravirine resistance requires at least two mutations [84] for it to have the highest genetic barrier in its class, and this is the reason why it is commonly used and combined with other drugs [85]. 

Another mutation, E138K, was associated with the virological failure of some patients receiving rilpivirine. Above all, this mutation is also responsible for some cross-resistance to etravirine, efavirenz, and nevirapine [86,87]. Among all, doravirin is approved for use in patients who are antiretroviral-naïve, as well as patients who have reached a stable virologic suppression [88]. It has a high genetic barrier to resistance with activity also held in the presence of a single NNRTI mutation, such as K103N, Y181C, and G190A [89].

Some relevant mutations in HIV-2 strains confer strong resistance to NNRTIs, such as V106I, E138A, and G190A [89].

## 7. PIs Drugs Resistance

PIs have high genetic barriers to resistance; they act as competitive inhibitors in the active site of HIV-1 protease that usually prevents the enzyme from processing the Gag and Gag/Pol polyprotein precursors necessary for viral maturation [90].

In subtype B viruses, resistance mutations at codons 33, 34, 58, 63, 73, 71, 77, and 84 are more common than in subtype F or C viruses, whereas those at codons 20, 36, and 89 are less common. In subtype F viruses, resistance mutations are more common at codons 10, 20, 35, 36, 48, 74, 57, 82, and 89, whereas they are less common at codons 47 and 93. In subtype C viruses, the frequency of resistance mutations is higher at codons 20, 36, 89, and 93 and lower at codons 10, 30, 43, 46, and 74 [91].

PI-resistance mutations are different: there are major ones and accessory ones, which both act by reducing susceptibility to one or more PIs. The accessory PI-resistance mutations, however, act in combination with the major PI-resistance mutations [54].

Between the different major mutations, such as D30N, V32I, M46IL, G48VM, I50VL, I54VTALM, L76V, V82ATFS, I84V, N88S, and L90M, some work by causing a high-level resistance to just one PI, while others by reducing susceptibility to two or more PIs. For example, D30N and I50L act, respectively, on nelfinavir and atazanavir. V82M is the mutation frequently developed in subtype G viruses [92]. Most PI-resistant viruses also require one or more accessory protease mutations and one or more compensatory gag cleavage-site mutations [93,94]. The requirement for multiple accessory mutations outside of protease probably contributes to the high genetic barrier to resistance of boosted PIs.

Three to four mutations are required for the development of a high-level lopinavir/r resistance and even more for a high-level darunavir/r resistance, showing that these two drugs have the highest genetic barriers to resistance [95,96].

Several studies suggest that atazanavir has a lower genetic barrier to resistance than lopinavir and darunavir. PI-resistance mutations develop less frequently in patients receiving an initial lopinavir-, atazanavir-, or darunavir-containing regimen than in patients receiving NRTI/NNRTI-containing regimens [97,98,99,100,101,102,103]. 

Moreover, it has been shown that administering protease inhibitors as monotherapy increases the chance of developing multiple resistance mutations in the protease gene with their continued use.

In particular, it has been demonstrated that prolonged monotherapy with a high dose of first-generation PI, such as saquinavir (58 weeks), resulted in multiple mutations in the protease gene as V28A.

This relatively high rate of mutations following saquinavir therapy was likely due to the fact that the drug does not report the same antiviral effects when given as monotherapy, compared to last-generation PIs, such as darunavir, without ritonavir booster and for prolonged periods of time [104].

HIV-1 subtype A and subtype B were shown to develop different mutations in the protease gene when saquinavir was used [105].

Among the PI drugs, fosamprenavir, a prodrug of amprenavir, was approved in 2016 for use in children aged 4 weeks to 18 years in the United States. The overall types and patterns of treatment-emergent, viral resistance–associated mutations from children receiving fosamprenavir/ritonavir-based regimens were similar to the resistance profiles seen in adults receiving fosamprenavir [106]. Moreover, amprenavir was also shown to possess activity against the hepatitis C virus and inhibit the migration of human hepatocarcinoma cells and the growth of xenografts [107].

HIV-2 shows partial resistance to nelfinavir, ritonavir, indinavir, atazanavir, and tipranavir. There is little specific information on mutational pathways leading to HIV-2 resistance to protease inhibitors. Some evidence has been collected from clinical and in vitro studies. Thus, 154M is a mutation frequently selected in the presence of nelfinavir or indinavir, while 182L seems to be associated with tipranavir resistance, and the combination of 154M, 184V, and L90M mutations confers high-level resistance to most protease inhibitors, such as saquinavir, lopinavir, and darunavir. The presence of I54M, together with I82F, has also been associated with lopinavir therapy failure in treated patients [17].

## 8. INSTIs Drugs Resistance

INSTIs block the action of the integrase viral enzyme (responsible for the insertion of the HIV-1 genome into the host DNA) by impeding the correct positioning of viral DNA at the active site of the enzyme and by binding to the catalytic metal cations inside the retroviral IN active site [54]. Raltegravir resistance occurs by three main, occasionally overlapping, mutational pathways: N155H ± E92Q; Q148H/R/K ± G140S/A; Y143C/R [108,109]. Each of these pairs of mutations is often accompanied by other accessory mutations [108]. N155H, Q148R, Y143R, and other mutations have been shown to reduce raltegravir susceptibility. It is very important to consider that except for Y143C/R, most raltegravir-resistance mutations confer cross-resistance to elvitegravir.

Likewise, most elvitegravir-resistance mutations are the cause of raltegravir cross-resistance. Dolutegravir requires a Q148 mutation in combination with E138 ± G140 since it has a higher genetic barrier to resistance than raltegravir and elvitegravir, requiring a Q148 mutation in combination with E138 ± G140 [85]. However, there are other mutations, including N155H in combination with Q148, that appear to increase the risk of developing resistance to dolutegravir.

The fact that a single mutation can reduce raltegravir (and elvitegravir) susceptibility more than 10-fold suggests that these inhibitors have a low genetic barrier to resistance [110,111].

Significant variability in INSTI-associated mutations can influence integrase activity and viral resistance among HIV-1 subtypes [112].

Resistance-associated mutations implicated in raltegravir and elvitegravir resistance, such as E92Q, Q148K/R/H, N155H, and E157Q, have been observed in HIV-1 subtype B virus and HIV-1 subtype CRF02_AG virus. In some patients infected with HIV-1 CRF02_AG, it was observed that having a G118R mutation meant much higher levels of raltegravir resistance than the ones observed in HIV-1 subtype B. On the contrary, HIV-O subtype B harboring Q-148R and N155H mutations has shown a higher resistance to dolutegravir rather than HIV-M subtype B [112].

Other polymorphic mutations, such as L74M, T97A, and E157E/G, were found in both HIV-1 B and non-B subtypes [112].

Dolutegravir resistance is related to different mutations according to different HIV-1 subtypes. Specifically, the G118R substitution was selected alone and in combination with H51Y in tissue culture selection experiments with CRF02_AG and subtype C viruses, but it was never selected in subtype B viruses [112].

Moreover, it has been found that HIV-1/non-M viruses are naturally susceptible to dolutegravir and raltegravir [112].

Little information is known about HIV-2–resistance pathways to approved integrase inhibitors. It is likely that resistance mutations of HIV-1 and HIV-2 are broadly the same.

In particular, not much is known about dolutegravir action against HIV-2, even if it works on raltegravir- and elvitegravir-resistant HIV-1 strains; moreover, raltegravir and elvitegravir have a low genetic barrier and show a high cross-resistance.

There are three major mutational pathways leading to high-level resistance to raltegravir and elvitegravir in HIV-2. Their characteristic amino acid substitutions are (i) E92Q/Y143C or T97A/Y143C, (ii) Q148K, Q148R or the combination G140S/Q148R, and (iii) N155H or the combinations E92Q/N155H and T97A/N155H [17].

## 9. Entry Inhibitor Drugs Resistance

### 9.1. Chemokine Receptor 5 (CCR5) Antagonists

The small-molecule inhibitor maraviroc allosterically inhibits the binding of HIV-1 gp120 to the host CCR5 (R5) co-receptor. Its use is limited to the early phases of HIV infection because CCR5 tropic virus prevalence is very low in the advanced phase of the infection. Mutations seen in patients enrolled in the MOTIVATE trials include G11S + I26V, S18G + A22T, A19S + I26V, I20F + A25D + I26V, and I20F + Y21I in the V3 region. Mutation-conferring resistance to maraviroc affects the V3 loop region and involves mutations that result in increased affinity of gp120 to MVC-bound CCR5, enabling gp120 binding to CCR5 despite conformational changes from MVC binding [113]. Studies on maraviroc resistance patterns are limited [113,114]. 

### 9.2. Entry Inhibitor of gp41

Enfuvirtide, the only approved entry inhibitor, binds to gp41, preventing the creation of the entry pore for the capsid of the virus and keeping it out of the cell [115].

Enfuvirtide has a low genetic barrier [116,117]. Mutations that confer enfuvirtide resistance are localized in its binding site, gp41, in codons 36–45 [118,119,120]. In particular, when only one single mutation affects the site of the enfuvirtide, susceptibility is reduced by about 10-fold, while two mutations lead to a reduction of about 100-fold. The most common enfuvirtide mutations are G36DEV, V38EA, Q40H, N42T, and N43D [85]. However, in a recent study, various polymorphisms were observed in the envelope gp41 region. The major polymorphisms were R46K/M/Q, E137K, and S138A. R46K/M/Q and S138A were predominant in subtype CRF07_BC, and E137K was prevalent in subtype B [121].

## 10. Conclusions

HIV drug resistance is present all over the world. Approximately 38 million people across the globe live with HIV/AIDS, and of these, 36.2 million are adults, and 1.8 million are children (<15 years old). The different available studies concerning the emergence of HIV drug resistance are still in their infancy but pave the way to help in understanding the possible future steps to follow. Our work tried to highlight the large number of mutations present globally and underline that many drugs previously administered as part of ART during recent years have lost their efficacy, owing to viral resistance development, prompting new studies in this field to identify new drugs using other mechanisms to inhibit HIV replication.

ART has been shown to reduce HIV mortality, morbidity, and transmission. However, the presence of HIV subtypes with increasing drug resistance can compromise the effectiveness of antiretroviral drugs [85]. The different subtypes of HIV-1 and HIV-2 predominate in certain geographic areas, but their distribution is becoming increasingly heterogeneous as the pandemic progresses. As shown above, certain subtypes, in particular the resistant ones, may be associated with an increased risk of transmission and a faster progression to the development of AIDS.

The rise in HIV drug resistance is one of the greatest threats to global health that can result in millions of deaths. Diagnostic tools such as resistance testing should be considered an integral part of the plan to address this problem. Nevertheless, it is important to remember the limits of these tests since they only report mutations already known to be associated with drug resistance, while they are less interpretable when compared to new drugs or newer mutations. Plus, the interpretation of their results requires a wide expert panel. However, such testing, based on NGS sequencing, can be helpful in estimating the intrinsic replication competence of the virus [122], but resistance testing is currently only recommended for drug-experienced patients for whom therapy is not successful. Resistance testing should, instead, be applied to treatment-naïve patients to find any mutation resistance before beginning the treatment.

Although great progress has been made in monitoring population-level emergence and transmission of HIV drug resistance mutations, the mutated and resistant strains that reemerge under drug-induced selective pressure need to be effectively detected and preferentially extinguished to prevent the development of AIDS.

## Data Availability

The data presented in this study are available on request from the corresponding author.

## Supplementary Materials

The following supporting information can be downloaded at: https://www.mdpi.com/article/10.3390/microorganisms11010221/s1, Table S1: Drug resistance mutations.

## References

[1] Bbosa N., Kaleebu P., Ssemwanga D. (2019). HIV Subtype Diversity Worldwide. Curr. Opin. HIV AIDS.

[2] Gallo R.C., Montagnier L. (2003). The Discovery of HIV as the Cause of AIDS. N. Engl. J. Med..

[3] Montagnier L. (2010). 25 Years after HIV Discovery: Prospects for Cure and Vaccine. Virology.

[4] Vahlne A. (2009). A Historical Reflection on the Discovery of Human Retroviruses. Retrovirology.

[5] Hemelaar J., Gouws E., Ghys P.D., Osmanov S. (2006). Global and Regional Distribution of HIV-1 Genetic Subtypes and Recombinants in 2004. AIDS.

[6] Sharp P.M., Hahn B.H. (2011). Origins of HIV and the AIDS Pandemic. Cold Spring Harb. Perspect. Med..

[7] Vidal N., Peeters M., Mulanga-Kabeya C., Nzilambi N., Robertson D., Ilunga W., Sema H., Tshimanga K., Bongo B., Delaporte E. (2000). Unprecedented Degree of Human Immunodeficiency Virus Type 1 (HIV-1) Group M Genetic Diversity in the Democratic Republic of Congo Suggests That the HIV-1 Pandemic Originated in Central Africa. J. Virol..

[8] Casado G., Thomson M.M., Sierra M., Nájera R. (2005). Identification of a Novel HIV-1 Circulating ADG Intersubtype Recombinant Form (CRF19_cpx) in Cuba. J. Acquir. Immune Defic. Syndr..

[9] Ng K.T., Ong L.Y., Takebe Y., Kamarulzaman A., Tee K.K. (2012). Genome Sequence of a Novel HIV-1 Circulating Recombinant Form 54_01B from Malaysia. J. Virol..

[10] Song H., Giorgi E.E., Ganusov V.V., Cai F., Athreya G., Yoon H., Carja O., Hora B., Hraber P., Romero-Severson E. (2018). Tracking HIV-1 Recombination to Resolve Its Contribution to HIV-1 Evolution in Natural Infection. Nat. Commun..

[11] Osmanov S., Pattou C., Walker N., Schwardländer B., Esparza J. (2002). WHO-UNAIDS Network for HIV Isolation and Characterization Estimated Global Distribution and Regional Spread of HIV-1 Genetic Subtypes in the Year 2000. J. Acquir. Immune Defic. Syndr..

[12] Hanna G.J., Balaguera H.U., Freedberg K.A., Werner B.G., Steger Craven K.A., Craven D.E., D’Aquila R.T. (2003). Drug-Selected Resistance Mutations and Non-B Subtypes in Antiretroviral-Naive Adults with Established Human Immunodeficiency Virus Infection. J. Infect. Dis..

[13] Rhee S.-Y., Shafer R.W. (2018). Geographically-Stratified HIV-1 Group M Pol Subtype and Circulating Recombinant Form Sequences. Sci. Data.

[14] Gartner M.J., Roche M., Churchill M.J., Gorry P.R., Flynn J.K. (2020). Understanding the Mechanisms Driving the Spread of Subtype C HIV-1. EBioMedicine.

[15] Khan S., Zahid M., Qureshi M.A., Mughal M.N., Ujjan I.D. (2018). HIV-1 Genetic Diversity, Geographical Linkages and Antiretroviral Drug Resistance among Individuals from Pakistan. Arch. Virol..

[16] Korber B., Gaschen B., Yusim K., Thakallapally R., Kesmir C., Detours V. (2001). Evolutionary and Immunological Implications of Contemporary HIV-1 Variation. Br. Med. Bull..

[17] Menéndez-Arias L., Alvarez M. (2014). Antiretroviral Therapy and Drug Resistance in Human Immunodeficiency Virus Type 2 Infection. Antivir. Res..

[18] Lu H., Tang Y.-W. (2019). Myths in the Laboratory Diagnosis of HIV Infection. Emerg. Microbes Infect..

[19] Hurt C.B., Nelson J.A.E., Hightow-Weidman L.B., Miller W.C. (2017). Selecting an HIV Test: A Narrative Review for Clinicians and Researchers. Sex. Transm. Dis..

[20] Alexander T.S. (2016). Human Immunodeficiency Virus Diagnostic Testing: 30 Years of Evolution. Clin. Vaccine Immunol..

[21] Pitasi M.A., Patel S.N., Wesolowski L.G., Masciotra S., Luo W., Owen S.M., Delaney K.P. (2020). Performance of an Alternative Laboratory-Based HIV Diagnostic Testing Algorithm Using HIV-1 RNA Viral Load. Sex. Transm. Dis..

[22] Robertson M.M., Braunstein S.L., Hoover D.R., Li S., Nash D. (2019). Timeliness of Human Immunodeficiency Virus Diagnosis and Antiretroviral Treatment Initiation in the Era of Universal Testing and Treatment. J. Infect. Dis..

[23] Yang O.O., Cumberland W.G., Escobar R., Liao D., Chew K.W. (2017). Demographics and Natural History of HIV-1-Infected Spontaneous Controllers of Viremia. AIDS.

[24] Platten M., Linnemann R., Kümmerle T., Jung N., Wyen C., Ehren K., Gravemann S., Gillor D., Cornely O.A., Fischer J. (2014). Clinical Course and Quality of Care in ART-Naïve Patients Newly Presenting in a HIV Outpatient Clinic. Infection.

[25] Sneller M.C., Blazkova J., Justement J.S., Shi V., Kennedy B.D., Gittens K., Tolstenko J., McCormack G., Whitehead E.J., Schneck R.F. (2022). Combination Anti-HIV Antibodies Provide Sustained Virological Suppression. Nature.

[26] Merigan T.C. (1991). Treatment of AIDS with Combinations of Antiretroviral Agents. Am. J. Med..

[27] Nastri B.M., Zannella C., Folliero V., Rinaldi L., Restivo L., Stelitano D., Sperlongano R., Adinolfi L.E., Franci G. (2020). Editorial-Role of Highly Active Antiretroviral Therapy (HAART) for the COVID-19 Treatment. Eur. Rev. Med. Pharm. Sci..

[28] Sanna G., Madeddu S., Murgia G., Serreli G., Begala M., Caboni P., Incani A., Franci G., Galdiero M., Giliberti G. (2020). Potent and Selective Activity against Human Immunodeficiency Virus 1 (HIV-1) of *Thymelaea Hirsuta* Extracts. Viruses.

[29] Scott L.J. (2020). Dolutegravir/Lamivudine Single-Tablet Regimen: A Review in HIV-1 Infection. Drugs.

[30] Sarafianos S.G., Hughes S.H., Arnold E. (2004). Designing Anti-AIDS Drugs Targeting the Major Mechanism of HIV-1 RT Resistance to Nucleoside Analog Drugs. Int. J. Biochem. Cell Biol..

[31] Voshavar C. (2019). Protease Inhibitors for the Treatment of HIV/AIDS: Recent Advances and Future Challenges. Curr. Top. Med. Chem..

[32] Farady C.J., Craik C.S. (2010). Mechanisms of Macromolecular Protease Inhibitors. ChemBiochem.

[33] Ghosh A.K., Osswald H.L., Prato G. (2016). Recent Progress in the Development of HIV-1 Protease Inhibitors for the Treatment of HIV/AIDS. J. Med. Chem..

[34] Brik A., Wong C.-H. (2003). HIV-1 Protease: Mechanism and Drug Discovery. Org. Biomol. Chem..

[35] Anstett K., Brenner B., Mesplede T., Wainberg M.A. (2017). HIV Drug Resistance against Strand Transfer Integrase Inhibitors. Retrovirology.

[36] Delelis O., Carayon K., Saïb A., Deprez E., Mouscadet J.-F. (2008). Integrase and Integration: Biochemical Activities of HIV-1 Integrase. Retrovirology.

[37] Quashie P.K., Mesplède T., Wainberg M.A. (2013). HIV Drug Resistance and the Advent of Integrase Inhibitors. Curr. Infect. Dis. Rep..

[38] Esté J.A., Telenti A. (2007). HIV Entry Inhibitors. Lancet.

[39] Pugach P., Ketas T.J., Michael E., Moore J.P. (2008). Neutralizing Antibody and Anti-Retroviral Drug Sensitivities of HIV-1 Isolates Resistant to Small Molecule CCR5 Inhibitors. Virology.

[40] Qian K., Morris-Natschke S.L., Lee K.-H. (2009). HIV Entry Inhibitors and Their Potential in HIV Therapy. Med. Res. Rev..

[41] Dvory-Sobol H., Shaik N., Callebaut C., Rhee M.S. (2022). Lenacapavir: A First-in-Class HIV-1 Capsid Inhibitor. Curr. Opin. HIV AIDS.

[42] Diamond T.L., Ngo W., Xu M., Goh S.L., Rodriguez S., Lai M.-T., Asante-Appiah E., Grobler J.A. (2022). Islatravir Has a High Barrier to Resistance and Exhibits a Differentiated Resistance Profile from Approved Nucleoside Reverse Transcriptase Inhibitors (NRTIs). Antimicrob. Agents Chemother..

[43] Cane P.A. (2005). Stability of Transmitted Drug-Resistant HIV-1 Species. Curr. Opin. Infect. Dis..

[44] Kitayimbwa J.M., Mugisha J.Y.T., Saenz R.A. (2016). Estimation of the HIV-1 Backward Mutation Rate from Transmitted Drug-Resistant Strains. Popul. Biol..

[45] Abram M.E., Ferris A.L., Shao W., Alvord W.G., Hughes S.H. (2010). Nature, Position, and Frequency of Mutations Made in a Single Cycle of HIV-1 Replication. J. Virol..

[46] Mansky L.M. (2002). HIV Mutagenesis and the Evolution of Antiretroviral Drug Resistance. Drug Resist. Updat..

[47] Keele B.F., Giorgi E.E., Salazar-Gonzalez J.F., Decker J.M., Pham K.T., Salazar M.G., Sun C., Grayson T., Wang S., Li H. (2008). Identification and Characterization of Transmitted and Early Founder Virus Envelopes in Primary HIV-1 Infection. Proc. Natl. Acad. Sci. USA.

[48] Nasir A., Dimitrijevic M., Romero-Severson E., Leitner T. (2021). Large Evolutionary Rate Heterogeneity among and within HIV-1 Subtypes and CRFs. Viruses.

[49] Levy D.N., Aldrovandi G.M., Kutsch O., Shaw G.M. (2004). Dynamics of HIV-1 Recombination in Its Natural Target Cells. Proc. Natl. Acad. Sci. USA.

[50] Hammer S.M., Saag M.S., Schechter M., Montaner J.S.G., Schooley R.T., Jacobsen D.M., Thompson M.A., Carpenter C.C.J., Fischl M.A., Gazzard B.G. (2006). Treatment for Adult HIV Infection: 2006 Recommendations of the International AIDS Society-USA Panel. JAMA.

[51] Coffin J.M. (1995). HIV Population Dynamics in Vivo: Implications for Genetic Variation, Pathogenesis, and Therapy. Science.

[52] Miller R.L., Ponte R., Jones B.R., Kinloch N.N., Omondi F.H., Jenabian M.-A., Dupuy F.P., Fromentin R., Brassard P., Mehraj V. (2019). HIV Diversity and Genetic Compartmentalization in Blood and Testes during Suppressive Antiretroviral Therapy. J. Virol..

[53] Perelson A.S., Ribeiro R.M. (2013). Modeling the Within-Host Dynamics of HIV Infection. BMC Biol..

[54] Tang M.W., Shafer R.W. (2012). HIV-1 Antiretroviral Resistance: Scientific Principles and Clinical Applications. Drugs.

[55] Sun Z., Lan Y., Liang S., Wang J., Ni M., Zhang X., Yu F., Chen M., Zhang H., Yan L. (2022). Prevalence of Doravirine Cross-Resistance in HIV-Infected Adults Who Failed First-Line ART in China, 2014-18. J. Antimicrob. Chemother..

[56] Novak R.M., Chen L., MacArthur R.D., Baxter J.D., Huppler Hullsiek K., Peng G., Xiang Y., Henely C., Schmetter B., Uy J. (2005). Prevalence of Antiretroviral Drug Resistance Mutations in Chronically HIV-Infected, Treatment-Naive Patients: Implications for Routine Resistance Screening before Initiation of Antiretroviral Therapy. Clin. Infect. Dis..

[57] Wensing A.M.J., van de Vijver D.A., Angarano G., Asjö B., Balotta C., Boeri E., Camacho R., Chaix M.-L., Costagliola D., De Luca A. (2005). Prevalence of Drug-Resistant HIV-1 Variants in Untreated Individuals in Europe: Implications for Clinical Management. J. Infect. Dis..

[58] Brenner B.G., Coutsinos D. (2009). The K65R Mutation in HIV-1 Reverse Transcriptase: Genetic Barriers, Resistance Profile and Clinical Implications. HIV.

[59] Ross L., Elion R., Lanier R., Dejesus E., Cohen C., Redfield R.R., Gathe J.C., Hsu R.K., Yau L., Paulsen D. (2009). Modulation of K65R Selection by Zidovudine Inclusion: Analysis of HIV Resistance Selection in Subjects with Virologic Failure Receiving Once-Daily Abacavir/Lamivudine/Zidovudine and Tenofovir DF (Study COL40263). AIDS Res. Hum. Retrovir..

[60] Bazmi H.Z., Hammond J.L., Cavalcanti S.C., Chu C.K., Schinazi R.F., Mellors J.W. (2000). In Vitro Selection of Mutations in the Human Immunodeficiency Virus Type 1 Reverse Transcriptase That Decrease Susceptibility to (-)-Beta-D-Dioxolane-Guanosine and Suppress Resistance to 3′-Azido-3′-Deoxythymidine. Antimicrob. Agents Chemother..

[61] Cilento M.E., Reeve A.B., Michailidis E., Ilina T.V., Nagy E., Mitsuya H., Parniak M.A., Tedbury P.R., Sarafianos S.G. (2021). Development of Human Immunodeficiency Virus Type 1 Resistance to 4′-Ethynyl-2-Fluoro-2′-Deoxyadenosine Starting with Wild-Type or Nucleoside Reverse Transcriptase Inhibitor-Resistant Strains. Antimicrob. Agents Chemother..

[62] Miller M.D. (2004). K65R, TAMs and Tenofovir. AIDS Rev..

[63] Luo X.-L., Mo L., Su G.-S., Huang J.-P., Wu J.-Y., Su H.-Z., Huang W.-H., Luo S., Ni Z.-Y. (2019). Incidence and Types of HIV-1 Drug Resistance Mutation among Patients Failing First-Line Antiretroviral Therapy. J. Pharm. Sci..

[64] McColl D.J., Chappey C., Parkin N.T., Miller M.D. (2008). Prevalence, Genotypic Associations and Phenotypic Characterization of K65R, L74V and Other HIV-1 RT Resistance Mutations in a Commercial Database. Antivir. Ther..

[65] Pozniak A. (2008). Tenofovir: What Have over 1 Million Years of Patient Experience Taught Us?. Int. J. Clin. Pract..

[66] Wolf K., Walter H., Beerenwinkel N., Keulen W., Kaiser R., Hoffmann D., Lengauer T., Selbig J., Vandamme A.-M., Korn K. (2003). Tenofovir Resistance and Resensitization. Antimicrob. Agents Chemother..

[67] Lambert-Niclot S., Charpentier C., Storto A., Fofana D., Soulie C., Fourati S., Wirden M., Morand-Joubert L., Masquelier B., Flandre P. (2014). Rilpivirine, Emtricitabine and Tenofovir Resistance in HIV-1-Infected Rilpivirine-Naive Patients Failing Antiretroviral Therapy. J. Antimicrob. Chemother..

[68] Ferrer E., Niubo J., Crespo M., Gatell J.M., Sanz J., Veloso S., Llibre J.M., Barrufet P., Sanchez P., Podzamczer D. (2007). Genotypic Resistance in HIV-Infected Naive Patients Receiving Abacavir plus Lamivudine and Efavirenz. J. Acquir. Immune Defic. Syndr..

[69] Galindo J., Amariles P., Mueses-Marín H.F., Hincapié J.A., González-Avendaño S., Galindo-Orrego X. (2016). Effectiveness and Safety of Generic Version of Abacavir/Lamivudine and Efavirenz in Treatment Naïve HIV-Infected Patients: A Nonrandomized, Open-Label, Phase IV Study in Cali-Colombia, 2011-2012. BMC Infect. Dis..

[70] Karkashadze E., Dvali N., Bolokadze N., Sharvadze L., Gabunia P., Karchava M., Tchelidze T., Tsertsvadze T., DeHovitz J., Del Rio C. (2019). Epidemiology of Human Immunodeficiency Virus (HIV) Drug Resistance in HIV Patients with Virologic Failure of First-Line Therapy in the Country of Georgia. J. Med. Virol..

[71] Ayitewala A., Kyeyune F., Ainembabazi P., Nabulime E., Kato C.D., Nankya I. (2020). Comparison of HIV Drug Resistance Profiles across HIV-1 Subtypes A and D for Patients Receiving a Tenofovir-Based and Zidovudine-Based First Line Regimens in Uganda. AIDS Res..

[72] Kisic M., Mendieta J., Puertas M.C., Parera M., Martínez M.A., Martinez-Picado J., Menéndez-Arias L. (2008). Mechanistic Basis of Zidovudine Hypersusceptibility and Lamivudine Resistance Conferred by the Deletion of Codon 69 in the HIV-1 Reverse Transcriptase Coding Region. J. Mol. Biol..

[73] Ren J., Stammers D.K. (2008). Structural Basis for Drug Resistance Mechanisms for Non-Nucleoside Inhibitors of HIV Reverse Transcriptase. Virus Res..

[74] Antinori A., Zaccarelli M., Cingolani A., Forbici F., Rizzo M.G., Trotta M.P., Di Giambenedetto S., Narciso P., Ammassari A., Girardi E. (2002). Cross-Resistance among Nonnucleoside Reverse Transcriptase Inhibitors Limits Recycling Efavirenz after Nevirapine Failure. AIDS Res. Hum. Retrovir..

[75] Ruxrungtham K., Pedro R.J., Latiff G.H., Conradie F., Domingo P., Lupo S., Pumpradit W., Vingerhoets J.H., Peeters M., Peeters I. (2008). Impact of Reverse Transcriptase Resistance on the Efficacy of TMC125 (Etravirine) with Two Nucleoside Reverse Transcriptase Inhibitors in Protease Inhibitor-Naïve, Nonnucleoside Reverse Transcriptase Inhibitor-Experienced Patients: Study TMC125-C227. HIV Med..

[76] Varghese V., Shahriar R., Rhee S.-Y., Liu T., Simen B.B., Egholm M., Hanczaruk B., Blake L.A., Gharizadeh B., Babrzadeh F. (2009). Minority Variants Associated with Transmitted and Acquired HIV-1 Nonnucleoside Reverse Transcriptase Inhibitor Resistance: Implications for the Use of Second-Generation Nonnucleoside Reverse Transcriptase Inhibitors. J. Acquir. Immune Defic. Syndr..

[77] Melikian G.L., Rhee S.-Y., Varghese V., Porter D., White K., Taylor J., Towner W., Troia P., Burack J., Dejesus E. (2014). Non-Nucleoside Reverse Transcriptase Inhibitor (NNRTI) Cross-Resistance: Implications for Preclinical Evaluation of Novel NNRTIs and Clinical Genotypic Resistance Testing. J. Antimicrob. Chemother..

[78] Brenner B., Turner D., Oliveira M., Moisi D., Detorio M., Carobene M., Marlink R.G., Schapiro J., Roger M., Wainberg M.A. (2003). A V106M Mutation in HIV-1 Clade C Viruses Exposed to Efavirenz Confers Cross-Resistance to Non-Nucleoside Reverse Transcriptase Inhibitors. AIDS.

[79] Kolomeets A.N., Varghese V., Lemey P., Bobkova M.R., Shafer R.W. (2014). A Uniquely Prevalent Nonnucleoside Reverse Transcriptase Inhibitor Resistance Mutation in Russian Subtype A HIV-1 Viruses. AIDS.

[80] Sluis-Cremer N., Tachedjian G. (2008). Mechanisms of Inhibition of HIV Replication by Non-Nucleoside Reverse Transcriptase Inhibitors. Virus Res..

[81] Vingerhoets J., Tambuyzer L., Azijn H., Hoogstoel A., Nijs S., Peeters M., de Béthune M.-P., De Smedt G., Woodfall B., Picchio G. (2010). Resistance Profile of Etravirine: Combined Analysis of Baseline Genotypic and Phenotypic Data from the Randomized, Controlled Phase III Clinical Studies. AIDS.

[82] Cohen C.J., Andrade-Villanueva J., Clotet B., Fourie J., Johnson M.A., Ruxrungtham K., Wu H., Zorrilla C., Crauwels H., Rimsky L.T. (2011). Rilpivirine versus Efavirenz with Two Background Nucleoside or Nucleotide Reverse Transcriptase Inhibitors in Treatment-Naive Adults Infected with HIV-1 (THRIVE): A Phase 3, Randomised, Non-Inferiority Trial. Lancet.

[83] Rimsky L., Vingerhoets J., Van Eygen V., Eron J., Clotet B., Hoogstoel A., Boven K., Picchio G. (2012). Genotypic and Phenotypic Characterization of HIV-1 Isolates Obtained from Patients on Rilpivirine Therapy Experiencing Virologic Failure in the Phase 3 ECHO and THRIVE Studies: 48-Week Analysis. J. Acquir. Immune Defic. Syndr..

[84] Calza L., Magistrelli E., Colangeli V., Manfredi R., Borderi M., Rossi N., Conti M., Mancini R., Viale P. (2017). Dual Raltegravir-Etravirine Combination as Maintenance Regimen in Virologically Suppressed HIV-1-Infected Patients. AIDS Res. Hum. Retrovir..

[85] Clutter D.S., Jordan M.R., Bertagnolio S., Shafer R.W. (2016). HIV-1 Drug Resistance and Resistance Testing. Infect. Genet. Evol..

[86] Picchio G., Vingerhoets J., Tambuyzer L., Coakley E., Haddad M., Witek J. (2011). Short Communication Prevalence of Susceptibility to Etravirine by Genotype and Phenotype in Samples Received for Routine HIV Type 1 Resistance Testing in the United States. AIDS Res. Hum. Retrovir..

[87] Tambuyzer L., Vingerhoets J., Azijn H., Daems B., Nijs S., de Béthune M.-P., Picchio G. (2010). Characterization of Genotypic and Phenotypic Changes in HIV-1-Infected Patients with Virologic Failure on an Etravirine-Containing Regimen in the DUET-1 and DUET-2 Clinical Studies. AIDS Res. Hum. Retrovir..

[88] Rock A.E., Lerner J., Badowski M.E. (2020). Doravirine and Its Potential in the Treatment of HIV: An Evidence-Based Review of the Emerging Data. HIV.

[89] Stockdale A.J., Khoo S. (2022). Doravirine: Its Role in HIV Treatment. Curr. Opin. HIV AIDS.

[90] Ali A., Bandaranayake R.M., Cai Y., King N.M., Kolli M., Mittal S., Murzycki J.F., Nalam M.N.L., Nalivaika E.A., Özen A. (2010). Molecular Basis for Drug Resistance in HIV-1 Protease. Viruses.

[91] Munerato P., Sucupira M.C., Oliveros M.P.R., Janini L.M., de Souza D.F., Pereira A.A., Inocencio L.A., Diaz R.S. (2010). HIV Type 1 Antiretroviral Resistance Mutations in Subtypes B, C, and F in the City of São Paulo, Brazil. AIDS Res. Hum. Retrovir..

[92] Palma A.C., Covens K., Snoeck J., Vandamme A.-M., Camacho R.J., Van Laethem K. (2012). HIV-1 Protease Mutation 82M Contributes to Phenotypic Resistance to Protease Inhibitors in Subtype G. J. Antimicrob. Chemother..

[93] Foulkes-Murzycki J.E., Scott W.R.P., Schiffer C.A. (2007). Hydrophobic Sliding: A Possible Mechanism for Drug Resistance in Human Immunodeficiency Virus Type 1 Protease. Structure.

[94] Fun A., Wensing A.M.J., Verheyen J., Nijhuis M. (2012). Human Immunodeficiency Virus Gag and Protease: Partners in Resistance. Retrovirology.

[95] Doherty K.M., Nakka P., King B.M., Rhee S.-Y., Holmes S.P., Shafer R.W., Radhakrishnan M.L. (2011). A Multifaceted Analysis of HIV-1 Protease Multidrug Resistance Phenotypes. BMC Bioinform..

[96] Rhee S.-Y., Taylor J., Fessel W.J., Kaufman D., Towner W., Troia P., Ruane P., Hellinger J., Shirvani V., Zolopa A. (2010). HIV-1 Protease Mutations and Protease Inhibitor Cross-Resistance. Antimicrob. Agents Chemother..

[97] Barber T.J., Harrison L., Asboe D., Williams I., Kirk S., Gilson R., Bansi L., Pillay D., Dunn D. (2012). UK HIV Drug Resistance Database and UK Collaborative HIV Cohort (UK CHIC) Study Steering Committees Frequency and Patterns of Protease Gene Resistance Mutations in HIV-Infected Patients Treated with Lopinavir/Ritonavir as Their First Protease Inhibitor. J. Antimicrob. Chemother..

[98] Dolling D.I., Dunn D.T., Sutherland K.A., Pillay D., Mbisa J.L., Parry C.M., Post F.A., Sabin C.A., Cane P.A., UK HIV Drug Resistance Database (UKHDRD) (2013). Low Frequency of Genotypic Resistance in HIV-1-Infected Patients Failing an Atazanavir-Containing Regimen: A Clinical Cohort Study. J. Antimicrob. Chemother..

[99] El Bouzidi K., White E., Mbisa J.L., Phillips A., Mackie N., Pozniak A., Dunn D. (2014). Protease Mutations Emerging on Darunavir in Protease Inhibitor-Naïve and Experienced Patients in the UK. J. Int. AIDS Soc..

[100] Lathouwers E., De Meyer S., Dierynck I., Van de Casteele T., Lavreys L., de Béthune M.-P., Picchio G. (2011). Virological Characterization of Patients Failing Darunavir/Ritonavir or Lopinavir/Ritonavir Treatment in the ARTEMIS Study: 96-Week Analysis. Antivir. Ther..

[101] Molina J.-M., Cahn P., Grinsztejn B., Lazzarin A., Mills A., Saag M., Supparatpinyo K., Walmsley S., Crauwels H., Rimsky L.T. (2011). Rilpivirine versus Efavirenz with Tenofovir and Emtricitabine in Treatment-Naive Adults Infected with HIV-1 (ECHO): A Phase 3 Randomised Double-Blind Active-Controlled Trial. Lancet.

[102] Mollan K., Daar E.S., Sax P.E., Balamane M., Collier A.C., Fischl M.A., Lalama C.M., Bosch R.J., Tierney C., Katzenstein D. (2012). HIV-1 Amino Acid Changes among Participants with Virologic Failure: Associations with First-Line Efavirenz or Atazanavir plus Ritonavir and Disease Status. J. Infect. Dis..

[103] Van Zyl G.U., Liu T.F., Claassen M., Engelbrecht S., de Oliveira T., Preiser W., Wood N.T., Travers S., Shafer R.W. (2013). Trends in Genotypic HIV-1 Antiretroviral Resistance between 2006 and 2012 in South African Patients Receiving First- and Second-Line Antiretroviral Treatment Regimens. PLoS ONE.

[104] Montaner J.S.G., Schutz M., Schwartz R., Jayaweera D.T., Burnside A.F., Walmsley S., Saag M.S. (2006). Efficacy, Safety and Pharmacokinetics of Once-Daily Saquinavir Soft-Gelatin Capsule/Ritonavir in Antiretroviral-Naive, HIV-Infected Patients. MedGenMed.

[105] Jayaswal A., Mishra A., Mishra H., Shah K. (2014). Evaluation of Novel Saquinavir Analogs for Resistance Mutation Compatibility and Potential as an HIV-Protease Inhibitor Drug. Bioinformation.

[106] Ross L.L., Cotton M.F., Cassim H., Voronin E., Givens N., Sievers J., Cheng K.Y., APV29005 & APV20002 Pediatric Study Groups (2015). Treatment-Emergent Mutations and Resistance in HIV-Infected Children Treated with Fosamprenavir-Containing Antiretroviral Regimens. Open AIDS J..

[107] Esposito V., Verdina A., Manente L., Spugnini E.P., Viglietti R., Parrella R., Pagliano P., Parrella G., Galati R., De Luca A. (2013). Amprenavir Inhibits the Migration in Human Hepatocarcinoma Cell and the Growth of Xenografts. J. Cell Physiol..

[108] Blanco J.-L., Varghese V., Rhee S.-Y., Gatell J.M., Shafer R.W. (2011). HIV-1 Integrase Inhibitor Resistance and Its Clinical Implications. J. Infect. Dis..

[109] Geretti A.M., Armenia D., Ceccherini-Silberstein F. (2012). Emerging Patterns and Implications of HIV-1 Integrase Inhibitor Resistance. Curr. Opin. Infect. Dis..

[110] Cooper D.A., Steigbigel R.T., Gatell J.M., Rockstroh J.K., Katlama C., Yeni P., Lazzarin A., Clotet B., Kumar P.N., Eron J.E. (2008). Subgroup and Resistance Analyses of Raltegravir for Resistant HIV-1 Infection. N. Engl. J. Med..

[111] Lennox J.L., DeJesus E., Lazzarin A., Pollard R.B., Madruga J.V.R., Berger D.S., Zhao J., Xu X., Williams-Diaz A., Rodgers A.J. (2009). Safety and Efficacy of Raltegravir-Based versus Efavirenz-Based Combination Therapy in Treatment-Naive Patients with HIV-1 Infection: A Multicentre, Double-Blind Randomised Controlled Trial. Lancet.

[112] Han Y.-S., Mesplède T., Wainberg M.A. (2016). Differences among HIV-1 Subtypes in Drug Resistance against Integrase Inhibitors. Infect. Genet. Evol..

[113] Trkola A., Kuhmann S.E., Strizki J.M., Maxwell E., Ketas T., Morgan T., Pugach P., Xu S., Wojcik L., Tagat J. (2002). HIV-1 Escape from a Small Molecule, CCR5-Specific Entry Inhibitor Does Not Involve CXCR4 Use. Proc. Natl. Acad. Sci. USA.

[114] Moore J.P., Kuritzkes D.R. (2009). A Pièce de Resistance: How HIV-1 Escapes Small Molecule CCR5 Inhibitors. Curr. Opin. HIV AIDS.

[115] Lalezari J.P., Eron J.J., Carlson M., Cohen C., DeJesus E., Arduino R.C., Gallant J.E., Volberding P., Murphy R.L., Valentine F. (2003). A Phase II Clinical Study of the Long-Term Safety and Antiviral Activity of Enfuvirtide-Based Antiretroviral Therapy. AIDS.

[116] Cabrera C., Marfil S., García E., Martinez-Picado J., Bonjoch A., Bofill M., Moreno S., Ribera E., Domingo P., Clotet B. (2006). Genetic Evolution of Gp41 Reveals a Highly Exclusive Relationship between Codons 36, 38 and 43 in Gp41 under Long-Term Enfuvirtide-Containing Salvage Regimen. AIDS.

[117] Lu J., Deeks S.G., Hoh R., Beatty G., Kuritzkes B.A., Martin J.N., Kuritzkes D.R. (2006). Rapid Emergence of Enfuvirtide Resistance in HIV-1-Infected Patients: Results of a Clonal Analysis. J. Acquir. Immune Defic. Syndr..

[118] Marcelin A.-G., Reynes J., Yerly S., Ktorza N., Segondy M., Piot J.-C., Delfraissy J.-F., Kaiser L., Perrin L., Katlama C. (2004). Characterization of Genotypic Determinants in HR-1 and HR-2 Gp41 Domains in Individuals with Persistent HIV Viraemia under T-20. AIDS.

[119] Menzo S., Castagna A., Monachetti A., Hasson H., Danise A., Carini E., Bagnarelli P., Lazzarin A., Clementi M. (2004). Genotype and Phenotype Patterns of Human Immunodeficiency Virus Type 1 Resistance to Enfuvirtide during Long-Term Treatment. Antimicrob. Agents Chemother..

[120] Sista P.R., Melby T., Davison D., Jin L., Mosier S., Mink M., Nelson E.L., DeMasi R., Cammack N., Salgo M.P. (2004). Characterization of Determinants of Genotypic and Phenotypic Resistance to Enfuvirtide in Baseline and On-Treatment HIV-1 Isolates. AIDS.

[121] Chang L., Zhao J., Guo F., Ji H., Zhang L., Jiang X., Wang L. (2021). HIV-1 Gp41 Genetic Diversity and Enfuvirtide Resistance-Associated Mutations among Enfuvirtide-Naïve Patients in Southern China. Virus Res..

[122] Kageyama S., Amolong Hinay A., Telan E.F.O., Samonte G.M.J., Leano P.S.A., Tsuneki-Tokunaga A., Kanai K. (2019). Intrinsic Replication Competences of HIV Strains After Zidovudine/Lamivudine/Nevirapine Treatment in the Philippines. J. Int. Assoc. Provid. AIDS Care.

