# Supplementary material for: HIV and Drug-Resistant Subtypes
Source: Microorganisms. 2023 Jan 15;11(1):221. doi: 10.3390/microorganisms11010221 (PMC9861097; doi:10.3390/microorganisms11010221)
Supplement: Supplementary file 1 [file microorganisms-11-00221-s001.zip › microorganisms-1972714-supplementary.pdf]

Nucleoside reverse-transcriptase inhibitors (NRTIs)		Non Nucleoside reverse-transcriptase inhibitors (NRTIs)		Protease Inhibitors (PIs)		Integrase Strand Transfer Inhibitors (INSTIs)		Fusion Inhibitor of gp41
**HIV-1**	**HIV-2**	**HIV-1**	**HIV-2**	**HIV-1**	**HIV-2**	**HIV-1**	**HIV-2**	**HIV-1**
**Abacavir**<br>K65R [58,60-63]	**Abacavir**<br>Q151M [17]	**Delavirdine**<br>E138A[67]	**Delavirdine**<br>V106I[89]<br>E138A[89]<br>G190A[89]	**Atazanavir**<br>I50L[106]	**Atazanavir**	**Raltegravir**<br>N155H±E92Q [108-109]<br>Q148H/R/K± [108-109]<br>G140S/A[108-109]<br>Y143C/R[108-109]<br>E157Q[108-109]<br>G118R[108-109]	**Raltegravir**<br>E92Q/Y143C[17]<br>T97A/Y143C[17]<br>Q148K/R[17]<br>G140S/Q148R+N155H[17]<br>E92Q/N155H+T97A/N155H [17]	**Enfuvirtide**<br>G36DEV[118-120]<br>V38EA[118-120]<br>Q40H[118-120]<br>N42T[118-120]<br>N43D[118-120]<br>R46K/M/Q [118-120]<br>E137K[118-120]<br>S138A[118-120]
**Didanosine**<br>K65R [58,60-63]	**Didanosine**<br>Q151M [17]	**Efavirenz**<br>E138A[67]<br>L100I[77]<br>K101E/P[77]<br>K103N/S[77]	**Efavirenz**<br>V106I[89]<br>E138A[89]<br>G190A[89]	**Darunavir**	**Darunavir**<br>I84V[17]<br>L90M[17]<br>I54M[17]	**Elvitegravir**<br>Q148H/R/K[85]<br>G140S/A[110-111]<br>N155H[85]	**Elvitegravir**<br>E92Q/Y143C[17]<br>T97A/Y143C[17]<br>Q148K/R[17]<br>G140S/Q148R+N155H[17]	

		V106A/M [78]<br>Y188C/H/L [77]<br>G190A/S/E [77]<br>M230L[77]				E92Q[85]<br>E157Q[110-111]	E92Q/N155H+T97A/N155H [17]	
**Emtricitabine**<br>M184V/I [67]	**Emtricitabine**<br>Q151M[17]<br>M184I/V[17]<br>K65[17]	**Etravirine**<br>E138A[82-83]<br>L100I[82-82]<br>K101E/P[82-83]<br>Y181C/I/V [82-83]	**Etravirine**<br>V106I[89]<br>E138A[89]<br>G190A[89]	**Forsamprenavir**	**Forsamprenavir**	**Dolutegravir**<br>Q148+E138± [112]<br>G140[112]<br>N155H+Q148R [112]<br>G118R[112]<br>G118R-H51Y[112]	**Dolutegravir**	
**Lamivudine**<br>M184V/I [67]	**Lamivudine**<br>Q151M[17]	**Nevirapine**<br>E138A[86-87]<br>K101E/P[77]<br>K103N/S[77]<br>V106A/M [78]<br>Y181C/I/V [77]<br>Y188C/H/L [77]<br>G190A/S/E [77,80,81]<br>M230L[77]	**Nevirapine**<br>V106I[89]<br>E138A[89]<br>G190A[89]	**Indinavir**	**Indinavir**<br>I54M[17]			
**Stavudine**<br>M41L [30,59]<br>D67N [30,59]<br>K70R [30,59]<br>L210W [30,59]	**Stavudine**<br>Q151M[17]	**Rilpiravine**<br>E138A[84]<br>K101E/P[84]<br>K103N/S[84]<br>V106A/M	**Rilpiravine**<br>V106I[89]<br>E138A[89]<br>G190A[89]	**Nelfinavir**<br>D30N [92]	**Nelfinavir**<br>I54M[17]			

T215F/Y [30,59]<br>K219Q/E [30,59]		[84]<br>Y181C/I/V [84]<br>Y188C/H/L [84]<br>G190A/G/E[79]<br>M230L[84]<br>E138K[86-87]						
**Tenofovir**<br>K65R [66]<br>M184V/I [66]	**Tenofovir**<br>K65[17]			**Ritonavir**	**Ritonavir**<br>I54M[17]<br>I84V[17]<br>L90M[17]<br>I82F[17]			
**Zidovudine**<br>M41L[30,59]<br>D67N[30,59]<br>K70R[30,59]<br>L210W[30,59]<br>K219Q/E[30,59]<br>T215Y/F[30,59]	**Zidovudine**<br>Q151M[17]			**Saquinavir**<br>V28A[104]	**Saquinavir**<br>I84V[17]<br>L90M[17]<br>I54M[17]			
				**Tipranavir**	**Tipranavir**<br>I82L[17]			
